# Impact of antibiotic pharmacokinetics in urine on recurrent bacteriuria following treatment of complicated urinary tract infections

**DOI:** 10.1128/aac.00535-23

**Published:** 2023-09-28

**Authors:** David Melnick, Angela K. Talley, Vipul K. Gupta, Ian A. Critchley, Paul B. Eckburg, Kamal A. Hamed, Nivedita Bhatt, Gary Moore, Daren Austin, Christopher M. Rubino, Sujata M. Bhavnani, Paul G. Ambrose

**Affiliations:** 1 Spero Therapeutics, Inc., Cambridge, Massachusetts, USA; 2 Moore Computing Services, Inc., Little Rock, Arkansas, USA; 3 GlaxoSmithKline, London, UK; 4 Institute for Clinical Pharmacodynamics, Inc., Schenectady, New York, USA; Columbia University Irving Medical Center, New York, New York, USA

**Keywords:** urinary tract infection, bacteriuria, pharmacokinetics, tebipenem

## Abstract

The clinical relevance of bacteriuria following antibiotic treatment of complicated urinary tract infections in clinical trials remains controversial. We evaluated the impact of urine pharmacokinetics on the timing of recurrent bacteriuria in a recently completed trial that compared oral tebipenem pivoxil hydrobromide to intravenous ertapenem. The urinary clearance and urine dwell time of ertapenem were prolonged relative to tebipenem and were associated with a temporal difference in the repopulation of bladder urine with bacteria following treatment, potentially confounding the assessment of efficacy.

## INTRODUCTION

Discordance between clinical symptom resolution and recurrence of bacteriuria following antibiotic treatment is frequently reported across clinical trials of complicated urinary tract infections, including acute pyelonephritis (cUTI/AP), particularly in patients with underlying genitourinary abnormalities (1 – 14). Similarly, in clinical practice, recurrent post-treatment bacteriuria in the absence of clinical symptoms (i.e., asymptomatic bacteriuria) is frequent, and current treatment guidelines advocate against additional antibiotic therapy in this setting (15 – 19). In a systemic review and meta-analysis of recent cUTI/AP clinical trials, lower microbiological response rates were observed in patients treated with carbapenem antibiotics as compared with newer antibiotics, despite similar clinical response rates (20). Recurrence of bacteriuria may be due to the asymptomatic regrowth of persisting pathogens from protected sites in the urinary tract or to recolonization of the bladder urine by exogenous vaginal or urethral flora, as antibiotic concentrations decline to subinhibitory levels after treatment (20 – 29). To better understand this phenomenon and its clinical relevance, we evaluated the impact of urine pharmacokinetics (PK) on the timing of recurrent bacteriuria and microbiological response in a recently completed Phase 3 clinical trial (ADAPT-PO; ClinicalTrials.gov number, NCT03788967) comparing orally administered tebipenem pivoxil hydrobromide (TBP-PI-HBr 600 mg every 8 hours) to intravenously administered ertapenem (ETP 1,000 mg every 24 hours) in patients with cUTI/AP (3).

TBP-PI-HBr is an orally bioavailable carbapenem that is rapidly converted to tebipenem (TBP), the active moiety, after oral administration (30). Both TBP and ETP demonstrate broad spectrum *in vitro* activity against gram-positive and gram-negative pathogens, including fluoroquinolone-resistant and extended-spectrum β-lactamase-producing Enterobacterales isolates, with similar distributions of minimum inhibitory concentrations (31 – 36). However, the antibiotics differ in their PK profiles. TBP demonstrates low protein binding (free drug 58%) with a plasma half-life of 1 hour and a high fraction of the total dose excreted in urine (55%–60%) over 24 hours (37, 38). In contrast, ETP demonstrates high protein binding (free drug 5%) with a plasma half-life of 4–4.5 hours and a lower percentage excreted in urine (41–45%) over 24 hours (39 – 41). For the ADAPT-PO trial, the treatments were chosen to match plasma PK-pharmacodynamic (PK-PD) measures based on time-dependent microbicidal activity (3, 37 – 41). The mean duration of antibiotic treatment was similar (8.5–8.7 days). Clinical response rates [mean (%), treatment difference (TBP-ETP); 95% CI] were high and comparable at the end of treatment [EOT, ≥97.9%; 1.4 (−0.1, 3.4)], test of cure [TOC, ≥93.1%; −0.6 (−4.0, 2.8)], and late follow-up assessments [LFU, ≥88.6%; −1.5 (−5.7, 2.6)] (3). In contrast, microbiological response rates declined in both treatment arms after the EOT, with a larger difference between treatment arms at the TOC (median 7 days post-EOT) as compared with the EOT and LFU assessments [EOT, TBP 97.8%, ETP 96.2%; 1.5 (−0.8, 4.1); TOC, TBP 59.5%, ETP 63.5%; −4.5 (−10.8, 1.9); LFU, TBP 57.2%, ETP 58.2%; −1.5 (−7.9, 5.0)] (3). Furthermore, the pathogens recovered from urine at TOC were predominantly of the same genus and species as those recovered from individual patients at study entry and had similar MIC distribution, resistance determinants, and multi-locus sequence type as the baseline pathogens (data on file). These data support the conclusion that the microbiological failures at the TOC assessment were due to the regrowth of persisting pathogens. The larger difference in microbiological response rates at TOC as compared to EOT or LFU assessments was unexpected given the very similar MIC distributions (MIC_90_ vs Enterobacterales, 0.12 µg/mL) for both antibiotics. We posited that the difference in the timing of recurrent bacteriuria might be related to a difference in the rate of clearance of antibiotics from the urine after the completion of treatment.

**Fig 1:**
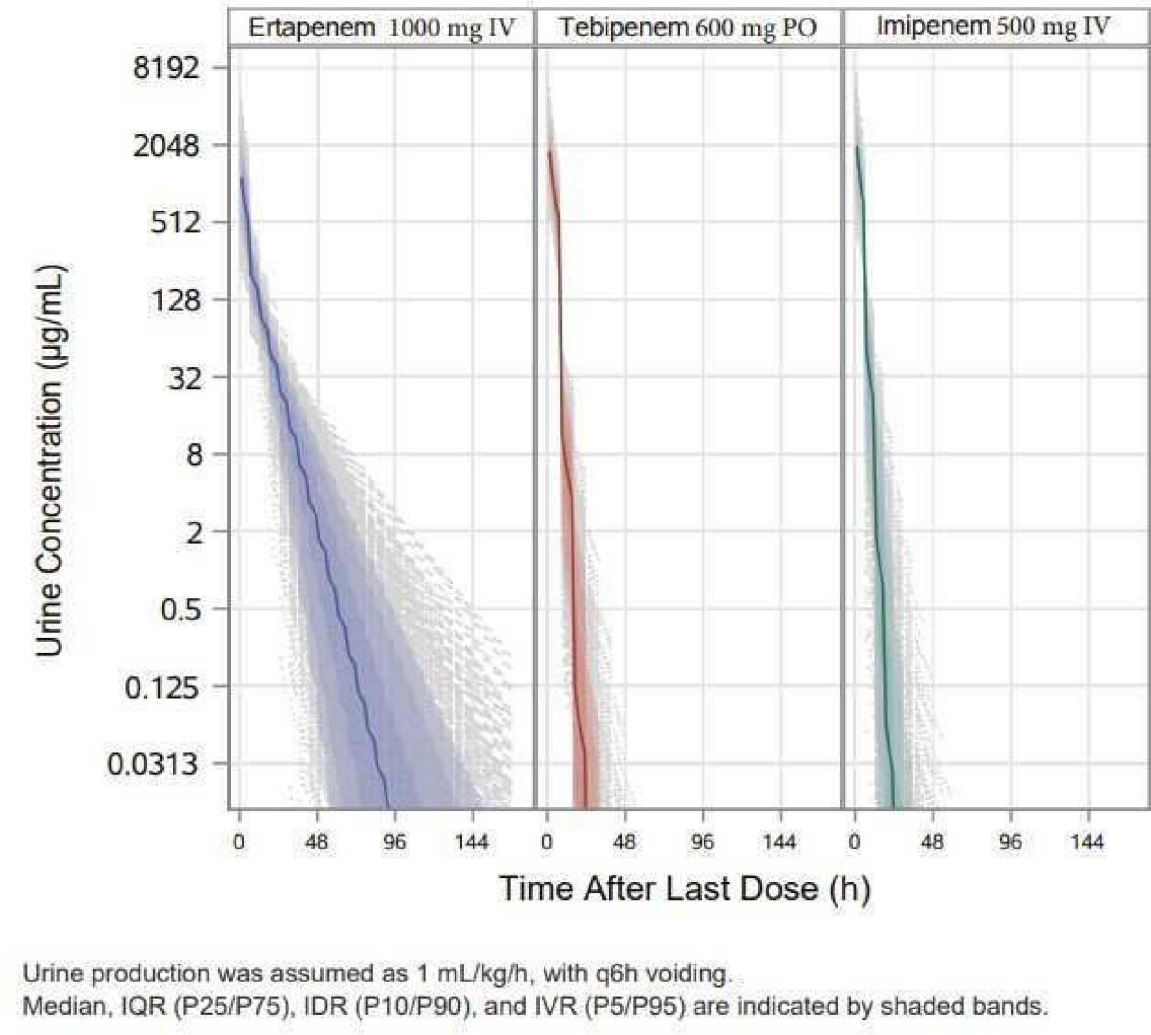
Simulated urinary concentrations for ETP, TBP, and imipenem from 30,000 subjects after the last dose of treatment: median, inter-quartile range (IQR, P25/75), inter-decile range (IDR, P10/P90), and inter-ventile range (IVR, P5/P95), with first 1,000 subjects shown.

The concentration-time profile in urine for TBP following oral administration of a 600 mg dose of TBP-PI-HBr was determined in healthy adult volunteers (38). The concentration-time profile in urine for ETP was modeled based on renal elimination of a 1,000 mg intravenous dose over time, as previously reported (39 – 41). Due to high protein binding, renal filtration and urinary elimination of ETP are considerably delayed as compared to TBP (Fig. 1). PK simulations demonstrated that urine concentrations of TBP fell below 0.12 µg/mL in virtually all patients within 24–36 hours. In contrast, ETP urine concentrations above 0.12 µg/mL were maintained in approximately 10% of the patients for 5 days and in some patients for more than a week after the last dose.

**Fig 2:**
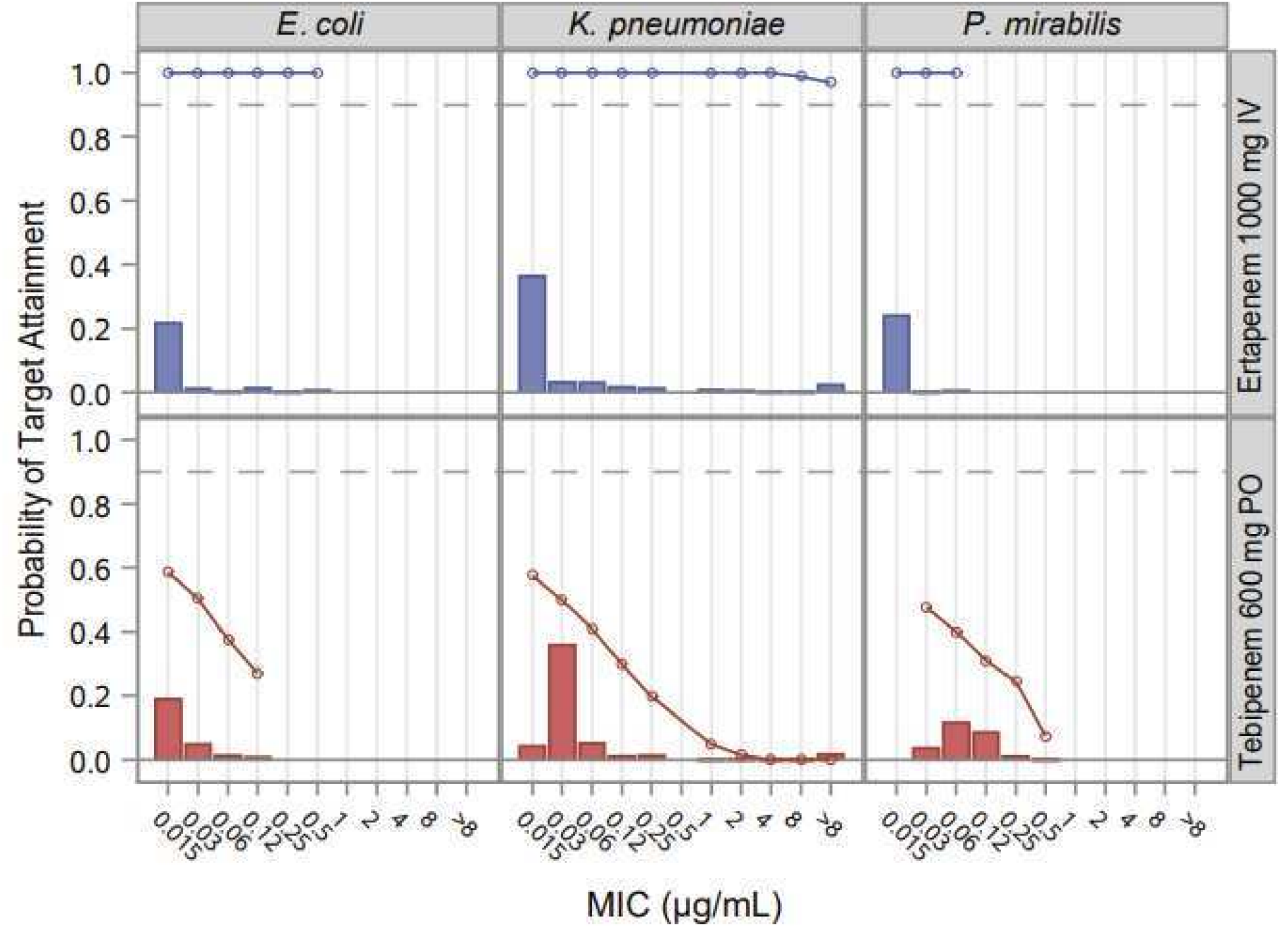
PTA in urine vs *Escherichia coli*, *Klebsiella pneumoniae,* and *Proteus mirabilis* in the week following cessation of treatment with TBP-PI-HBr 600 mg q8h and ETP 1,000 mg q24h. A target for PTA of 10% of the week above MIC was used. Pathogen frequency and MIC distributions from references 21, 31, with no correlation assumed.

**Fig 3:**
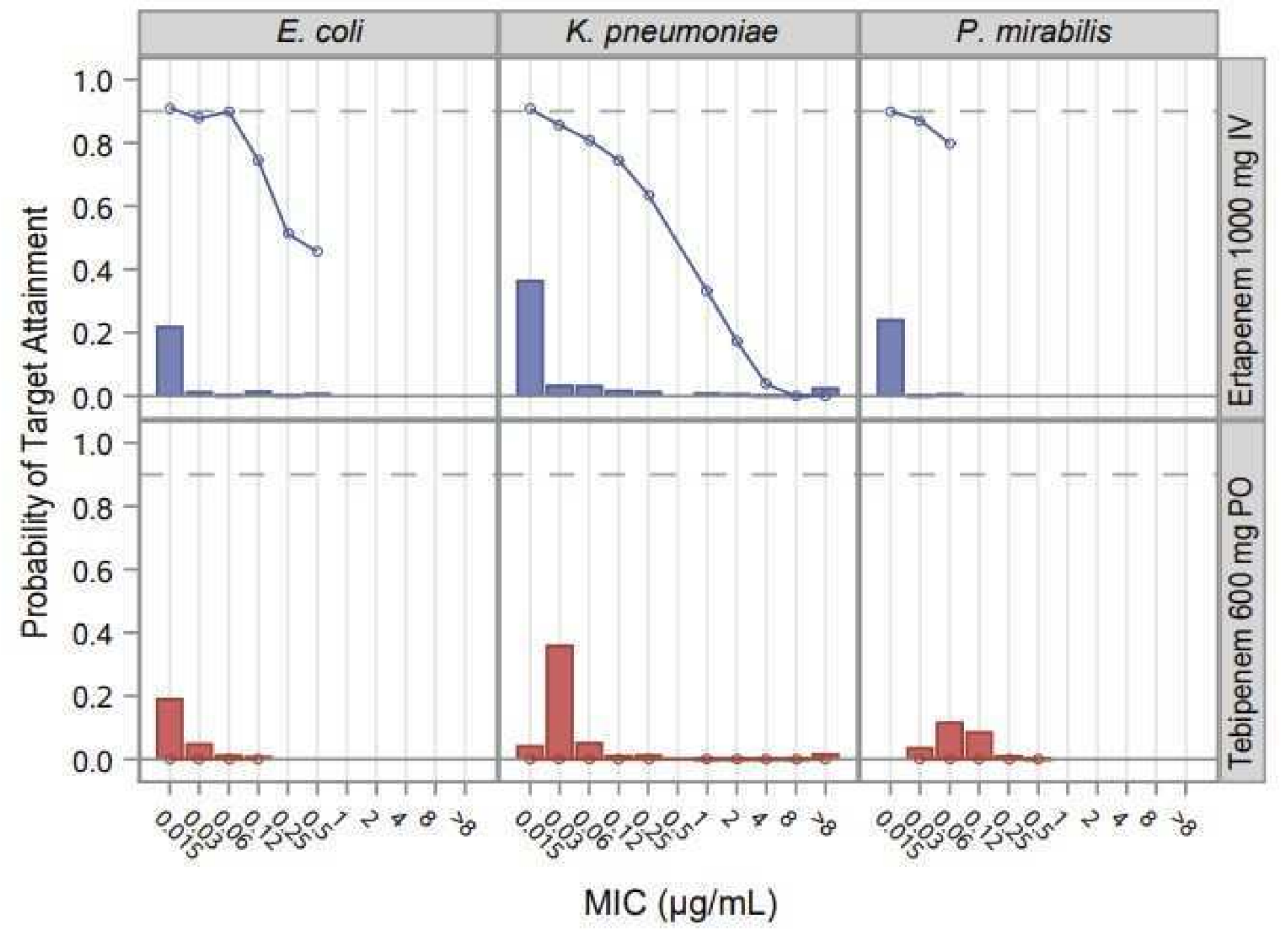
PTA in urine for *Escherichia coli*, *Klebsiella pneumoniae,* and *Proteus mirabilis* in the week following cessation of treatment with TBP-PI-HBr 600 mg q8h and ETP 1,000 mg q24h. A target for PTA of 30% of the week above MIC was used. Pathogen frequency and MIC distributions from references 21, 31, with no correlation assumed.

To further investigate the possible impact of the longer interval required for ETP to drop to subinhibitory urine levels in patients on the occurrence of bacteriuria at TOC assessment, Monte Carlo simulations were performed vs the MIC distribution of the most common Enterobacterales uropathogens (Fig. 2 and 3). The probability of target attainment (PTA) was evaluated by PK-PD population simulation methods. For each treatment regimen, 30,000 virtual subjects were generated. PK parameters were scaled by allometry. Urine concentrations were calculated based on the fraction excreted during the interval of interest (defined as 6 hours) and a constant assumed rate of urine production of 1 mL/kg/h. The fraction of time above MIC was calculated by subject-sampled MIC distribution across all subjects and summarized by drug, pathogen, and MIC.

Theoretical PK-PD targets of maintaining a urine concentration of TBP or ETP above a given MIC for 10% or 30% of the 7 days following the cessation of antibiotic therapy were interrogated. These theoretical targets were chosen to provide a quantitative comparison of the proportion of patients with inhibitory urine concentrations of TBP or ETP following the EOT. For a target of 10% urine time above MIC (T > MIC), based on the urine concentration-time profile, ETP demonstrated PTA > 99% at 0.12 µg/mL, the MIC_90_ for the Enterobacterales pathogens isolated at baseline. In contrast, TBP demonstrated a parallel PTA of ~30% for the 7 days following the EOT. For the theoretical target of maintaining urine concentrations above the MIC_90_ for 30% of the 7 days following the cessation of antibiotic therapy, ETP PTA was >70% compared with PTA of 0% for TBP.

It is plausible that repopulation of the bladder urine following treatment of a cUTI requires that antibiotic concentrations in urine drop to subinhibitory levels, allowing for persistent pathogens to resume planktonic growth (18, 19, 24, 25). Although urine PD requirements for suppression of bacteriuria have not been defined, the longer dwell time of ETP in urine after the last dose may contribute to the larger difference in microbiologic response seen at the TOC as compared to the EOT or LFU in this clinical trial. These hypothesis-generating data are being tested in an ongoing cUTI clinical trial comparing oral TBP-PI-HBr to intravenously administered imipenem-cilastatin, both with comparable urine PK (Fig. 1).
